# Editorial: New insights on the management of obesity with nutrition and physical activity

**DOI:** 10.3389/fnut.2023.1303965

**Published:** 2023-10-16

**Authors:** Hassane Zouhal, Claire Tourny, Kelly E. Johnson, Ismail Laher

**Affiliations:** ^1^Univ Rennes, M2S (Laboratoire Mouvement, Sport, Santé)-EA 1274, Rennes, France; ^2^Institut International des Sciences du Sport (2I2S), Irodouer, France; ^3^CETAPS UR 3832, University Rouen Normandy, Rouen, France; ^4^Department of Exercise and Sport Science, Coastal Carolina University, Conway, SC, United States; ^5^Department of Anesthesiology, Pharmacology and Therapeutics, University of British Columbia, Vancouver, BC, Canada

**Keywords:** obesity, overweight, children, adolescent, health, chronic diseases

## Introduction

Obesity is a rampant global epidemic that is associated with excessive accumulation of body fat, with psychological, social and somatic consequences affecting the quality of life (1–4). The World Health Organization (WHO) classified obesity as a chronic disease in 1997, declaring it as “the first non-infectious epidemic in history and a major problem in the world” (5). Obesity-related health concerns affecting children, adolescents, and adults have reached epidemic levels in both industrialized and developing countries (3, 4). The health risks and health care costs of childhood, adolescent, and adult obesity are considerable and include metabolic disorders, earlier puberty and menarche in girls, type 2 diabetes, hypertension, sleep apnea, adulthood obesity, and higher rates of mortality in young adults (6). Overweight and obesity among children, adolescents and adults are likely to be the result of complex interactions between genes, lifestyles, dietary habits, and socioeconomic factors. There is limited data on the determinants such as lifestyle behaviors, psycho-physiological factors, dietary habits, and familial factors leading to obesity and overweight (6).

An unhealthy lifestyle, including low levels of physical activity and increased intake of calorie-rich foods, is a key factor accelerating obesity (7). Implementation of appropriate nutrition and physical activity in individuals with overweight and/or obesity may improve the health of patients, especially those with adverse health consequences due to increased fat mass and adiposopathic metabolic consequences (8). Nutrition education and/or appropriate physical activity are important components in the management of individuals with obesity (9). However, despite the fact that a good diet and physical activity are known to confer protection against obesity, the molecular and cellular mechanisms that mediate the metabolic benefits of nutrition and/or physical activity remain unclear.

To clarify the effects of various aspects and interventions (nutrition, physical activity and others) in managing overweight and obesity in children, adolescents and adults, we launched a Frontiers Research Topic entitled “*New insights on the management of obesity with nutrition and physical activity”*. Accordingly, the goal of this Research Topic was to provide findings from original research and or review articles on the effects of physical activity and/or nutrition on the treatment of obesity.

## Summary of selected articles from this Research Topic

Twenty-three manuscripts were received for this Frontiers Research Topic. After rigorous review, nine articles were finally accepted for publication. The contributing 58 authors were from five countries, including Canada, China, Portugal, Spain and Saudi Arabia. This Research Topic received more than 11,000 views and downloads as of September 2023. The key contents and findings of each paper are as follows:

### Alsulami et al.

In this study, Alsulami et al., evaluated obesity prevalence, physical activity, and dietary practices among Saudi adults in the Makkah region of the Kingdom of Saudi Arabia (KSA).

The authors used a validated questionnaire, Arab Teens Lifestyle Study (ATLS), to evaluate the physical activities, sedentary behaviors, and nutritional habits in addition to demographic data. Their results show that overweight and obesity was prevalent in 32.8 and 23% of the population, respectively, and that sociodemographic factors were associated with obesity. However, focused intervention strategies are needed to overcome the prevalence of obesity in KSA.

### Chen et al.

The study by Chen et al. explored the associations of different types of unsaturated fatty acids (FAs) with overweight/obesity risk among the Chinese population. The study enrolled 8,742 subjects free of overweight/obesity at entry in the China Health and Nutrition Survey (CHNS) that were followed until 2015. Dietary unsaturated FAs were assessed by 3-day 24-h recalls with a weighing method in each wave. Cox regression models were used to obtain the hazard ratios (HRs) and 95% confidence intervals (CIs) for overweight/obesity risk associated with unsaturated FAs. The results demonstrated that higher dietary intake of monounsaturated FAs was associated with a lower overweight/obesity risk, which was mainly driven by dietary oleic acid from either plant or animal sources. Intakes of α-linolenic acid, n-6 polyunsaturated FAs and linoleic acid were related to a higher risk of overweight/obesity. The authors concluded that their results support consuming more monounsaturated FAs for maintaining a healthy body weight among the Chinese population.

### Lages et al.

The study of Lages et al. from Center for Innovative Care and Health Technology based in Polytechnic of Leira (Portugal) characterized the chronotype and determine its relation to the phenotype and dietary patterns of healthy patients and those with obesity.

The characterization of the chronotype and the circadian system as a phenotype is innovative and should be taken into consideration in patient-specific forms of obesity in order to develop targeted nutritional interventions. Dietary intake and sleep quality was assessed using validated questionnaires. Body composition was also assessed and blood samples taken to quantify circadian and metabolic biomarkers. This study has helped improve our understanding of the complex mechanisms underlying obesity.

### Ma et al.

The study by Ma et al. investigated how Chinese adolescents' lifestyles clustered into different lifestyle patterns, and analyzed the correlation between these patterns and adolescent overweight and obesity. The investigated respondents included 13,670 adolescents aged 13–18 from various administrative regions in China. The studied demonstrated that there was a coexistence of healthy behaviors and health-risk behaviors in the lifestyle clustering of Chinese adolescents. Low physical exercise and high intake of snacks and carbonated beverages were the most common. Physical exercise and health consciousness were the protective factors of overweight and obesity in adolescents.

### Salas-González et al.

The health risks of sedentary lifestyles are of increasing global concern, particularly in growing children. Many children and young adults seem addicted to electronic devices, are less likely to actively participate in sports and end up with less quality sleep, and it is likely that these traits will persist in adulthood. A study of Spanish primary school children from five geographic regions (*n* = 839 from 22 schools, aged 8–13 years, 51.1% girls) examined the relationship between 24 h movement guidelines (jointly self-reported by parents and children) and insulin resistance (calculated using the HOMA index). Plasma glucose and insulin levels were also measured. Those children (particularly girls) with insulin resistance had more sedentary lifestyles, while those who were more active had improved metabolic parameters—about half of the study group did not meet recommended movement guidelines, with better adherence to recommended total sleep times for this age group. A strength of the report by Salas-González et al. is studying a multitude of lifestyle factors (e.g., screen time, sleep time, movement), showing the importance of understanding the role of a combination of these coexisting choices.

### Shengyu et al.

The report by Shengyu et al. from Department of Economics and Management based in Changsha University (China) identified some risk factors affecting exercise behavior among overweight and obese people in China. This large-scale study of over 3,300 individuals considered the lifestyles of obese people. The study showed that the proportion of active physical activity among obese people was 25%. Groups with better sexual and reproductive health, higher levels of education and income were more likely to take part in sport. Obese people who lived in rural areas, were single or divorced, or in the 35–40 age bracket, had a significantly lower percentages of engagement in active physical activity. Health promotion programs for those who are obese need to be further strengthened and targeted to get closer to WHO recommendations.

### Tu et al.

The study by Tu et al., explored changes in physical characteristics in preschool children from 2000 to 2020, and forecast development trends over the next 10 years. The results show that the growth and nutritional status of Chinese preschoolers improved dramatically over the past 20 years, but that overweight and obesity remains. Overweight and obesity rates are expected to continue to increase rapidly over the next 10 years, particularly among boys, and effective measures should be taken to control the obesity epidemic.

### Wang et al.

The report by Manda Wang from Shunchang Li's group based in Chengdu Sport University adds to their other studies on the metabolic effects of MOTS-c, an endogenously produced regulator of metabolic homeostasis that stimulates glucose uptake and increases insulin sensitivity. The cardiovascular effects of MOTS-c include improved endothelial function, a hallmark of diabetes in humans and animal models. The study by Wang et al. reports on the cardiac structural and functional benefits of treating a rat model of type 2 diabetes with MOTS-c. Levels of endogenous MOTS-c are reduced in diabetes, and the results this study indicates that restoring MOTS-c levels with exogenously administered MOTS-c (i) repaired mitochondrial damage, (ii) restored cardiac systolic and diastolic function, (iii) improved gene regulation of fatty acid metabolism, immunoregulation, angiogenesis and apoptosis. These data provide the first molecular evidence that MOTS-c improves cardiovascular function in diabetes.

### Yu et al.

Yu et al. conducted a systematic review and network meta- analysis (NMA) by comparing adjuvant therapy with different nutritional supplements for overweight and obese adults, so as to provide a reference for clinical practice. The study show that nutritional supplementation lowered cardiovascular disease risk factors in overweight and obese patients. Probiotic supplementation might be the best intervention for blood glucose control; Vitamin D, probiotic + omega-3 have a better impact on improving lipid metabolism.

The editors of this volume are extremely grateful to the authors for their contributions and hard work sharing their research insights. We hope the readers of these papers will benefit from this Frontiers Research Topic and utilize the information herein to advance their scientific pursuits.

## Author contributions

HZ: Conceptualization, Writing—original draft, Writing—review and editing. CT: Conceptualization, Writing—original draft, Writing—review and editing. KJ: Conceptualization, Writing—original draft, Writing—review and editing. IL: Conceptualization, Writing—original draft, Writing—review and editing.
